# Sjögren-Larsson Syndrome: A Rare Presentation With Developmental Delay

**DOI:** 10.7759/cureus.35159

**Published:** 2023-02-18

**Authors:** Srilakshmi K J, Muhammad Daniyal Waheed, Saima Batool, Shaniah S Holder, Yadelys Rodriguez Reyes, Manisha Guntha

**Affiliations:** 1 Pediatrics, Dr. B. R. Ambedkar Medical College and Hospital, Bengaluru, IND; 2 Internal Medicine, Foundation University Medical College, Islamabad, PAK; 3 Internal Medicine, Hameed Latif Hospital, Lahore, PAK; 4 Medicine, American University of Barbados School of Medicine, Bridgetown, BRB; 5 Medicine, Universidad de Oriente Núcleo Anzoátegui, Barcelona, VEN; 6 Internal Medicine, Andhra Medical College, Visakhapatnam, IND

**Keywords:** congenital ichthyosis, spastic diplegia, developmental and behavioral delay, faldh, rare, sjögren–larsson syndrome, sjs

## Abstract

Sjögren-Larsson syndrome (SLS) is a rare, inherited disorder passed down through families in an autosomal recessive pattern. Its main characteristics are spastic diplegic paralysis, congenital ichthyotic hyperkeratosis, and mild-to-moderate mental retardation. Lack of activity of microsomal fatty aldehyde dehydrogenase (FALDH) or its complete absence is the primary cause of this syndrome, leading to the build-up of fatty aldehydes and fatty alcohols in the body, particularly in the skin. In order to provide the best care for patients, educating them about the management of dry skin and offering genetic counseling are essential. We hereby present a case of an eight-year-old patient with spastic diplegia, congenital ichthyosis, and intellectual disability diagnosed with SLS.

## Introduction

Sjögren-Larsson syndrome (SLS) is a rare autosomal recessive disorder caused by various mutations in the ALDH3A2 gene, leading to defective production of fatty aldehyde dehydrogenase (FALDH) [1]. This precipitates the inability to break down fatty alcohol molecules, allowing increased accumulation in the skin, neurological system, and eyes [1,2]. It presents with a triad of symptoms which consists of congenital ichthyosis, limb diplegia with or without spasticity, and intellectual disability [1]. SLS manifests initially with the development of ichthyosis, usually appearing in the early few months of life [2]. Neurological symptoms tend to begin within the first two years of life, and children present with developmental delays such as taking longer to learn how to crawl, speech issues, and in severe cases, a low IQ [2]. The rate of ocular symptoms reported in the literature has increased, with most persons displaying visual defects and photophobia due to retinal crystal formation [2]. The prevalence of SLS is notably higher in the pediatric population in Sweden, restricted to the Caucasian demographic, with an estimated prevalence of one in 250,000. However, an incidence rate has not been recorded internationally [3]. The first report of this condition was a case series including 28 patients in 1957 who had the triad of congenital ichthyosis, spastic diplegia or quadriplegia, and intellectual disability [4]. Other common manifestations of this condition include premature births, scoliosis, and short stature. The diagnosis of SLS is based on clinical findings and confirmed with imaging such as MRI and CT of the brain, which shows leukoencephalopathy [5]. There is no cure for SLS; therefore, symptomatic management is the course of treatment used. Speech and physical therapy for developmental delays and emollient use for dry, itchy skin is utilized to increase functionality and, subsequently, the quality of life in a person with SLS [5]. Although SLS is a congenital condition, many reports in the literature are of persons in their adult years; there is a paucity of literature regarding pediatric cases. Herein we report a case of an 8-year-old patient with congenital ichthyosis, intellectual disability, and spastic diplegia, who was found to have an elevated lipid-lactate peak on MRI spectroscopy of the brain and was subsequently diagnosed with SLS.

## Case presentation

An eight-year-old male child conceived through a consanguineous marriage was admitted to the Child Psychiatry Department of a tertiary care hospital with complaints of developmental delays and skin disorders since birth. The child was born at term (38 weeks) by cesarean section due to premature rupture of membranes. The child cried with a mild voice, but no other unusual conditions were reported at the time of birth except for when the child was unable to fold his hands due to a skin condition arising due to congenital ichthyosis. The child's weight at birth was 3.030 kg, and the length was 47 cm. His head circumference was 33 cm, and his blood group was B positive. Upon taking a detailed history, his parents informed him that he would respond to light and would move his head in the direction of the voice calling as a child. During the early days, special care was taken to keep the child's skin moist and alleviate any symptoms arising from ichthyosis by applying moisturizing creams (Cetaphil, Aveeno, Physiogel AI Lotion). The child rolled over in the seventh month and skipped the stage of crawling. He was able to sit with support at the age of 1 year and started standing without support at the age of 2 years. By the age of one, he was expressing his needs through actions and giving physical directions. The child started using single words at the age of 2 years. A neurological examination revealed a delay in reaching milestones and a mild intellectual disability, both of which were attributed to the child's chubby physical condition. Later on, in the course of development, the child presented with spasticity and stiffness in the lower limbs that was first observed during the latter part of the 11th or 12th month after birth, progressively worsening up to the time of presentation. A detailed evaluation of the child further revealed mild aphasia and increased tone in the lower limbs with slightly brisk deep tendon reflexes (DTR). His fundal and skeletal examinations were unremarkable, along with the tone and DTR in the upper limbs. However, his skin examination revealed pruritic scaly lesions all over the body, with their initial presentation starting in early infancy on the lower limbs. A wide array of hematological investigations were carried out (Table 1).

**Table 1:** Lab parameters. AST: Aspartate aminotransferase; SGOT: Serum glutamic-oxaloacetic transaminase; SGPT: Serum glutamic pyruvic transaminase; A-G Ratio: Albumin-Globulin Ratio; TIBC: Total iron binding capacity.

Particulars	Result	Unit	Reference
Total Bilirubin	0.6	mg/dl	0.3-1.3
Direct Bilirubin	0.2	mg/dl	0.2-0.4
Indirect Bilirubin	0.4	mg/dl	0.2-0.9
SGOT/AST (Serum) SGOT	35	U/L	10-37
SGPT/ALT (serum) SGPT	17	U/L	30-65
Alkaline Phosphatase	138	U/L	50-136
Total Protein	7.2	g/dl	6.4-8.2
Albumin	4.8	g/dl	3.4-5
Globulin	2.4	g/dl	
A-G Ratio	2		1-2
Urea	22.6	mg/dl	15-45
Creatinine	0.6	mg/dl	0.6-1.4
Ammonia	32.3	umol/L	11-32
Iron	105	ug/dl	65-175
TIBC	383	ug/dl	250-450
Ferritin	27	ng/ml	20-250
Transferin saturation	27.42	%	20-55
25 OH Vitamin D	19	ng/ml	20-40

**Figure 1:**
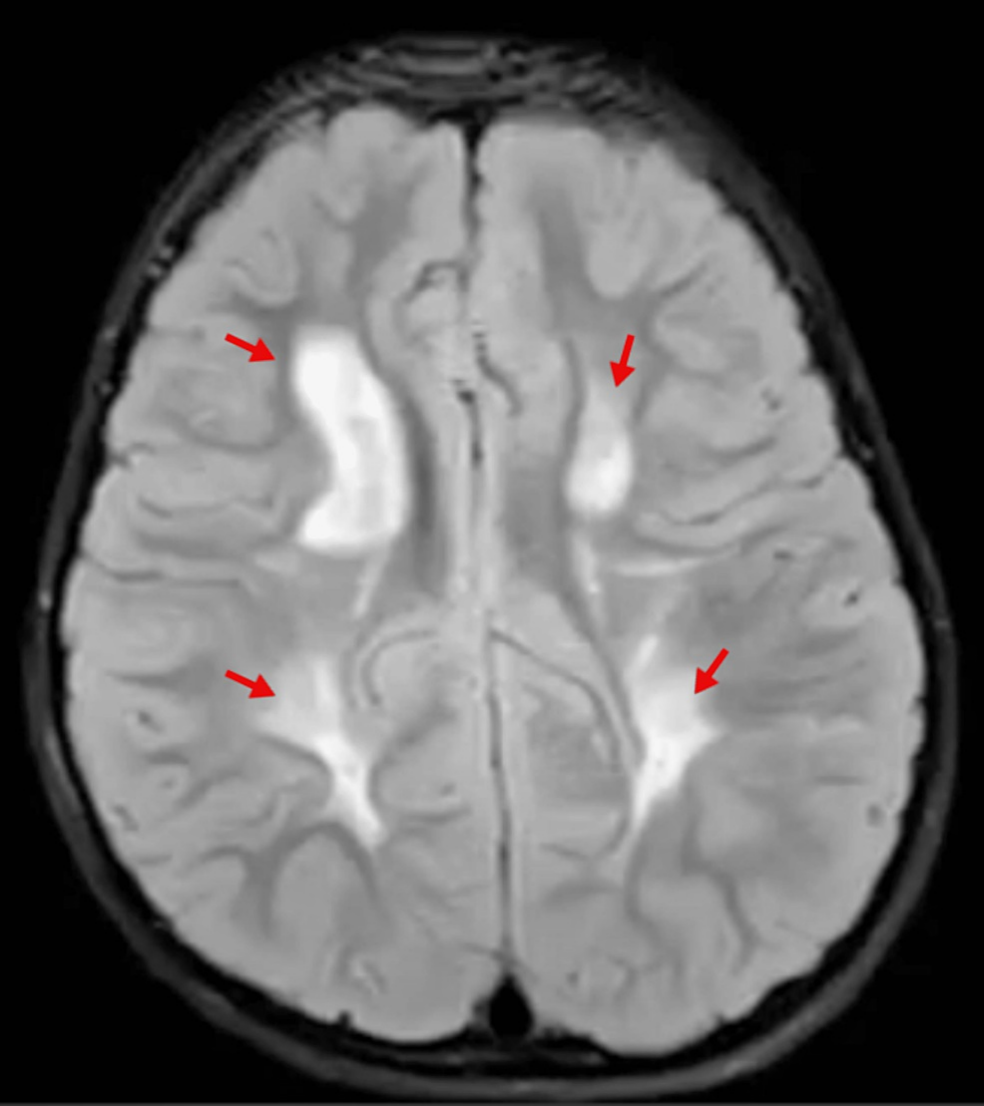
Bilateral fairly symmetrical confluent, periventricular T1 hypointense T2/Flair hyper intense (arrowheads).

His chest radiograph and electroencephalogram (EEG) were unremarkable. MRI brain plain and contrast was done. It showed the following observations: bilaterally symmetric, confluent T1 hypointense and T2 FLAIR hyperintense white matter signal abnormalities in the periventricular and deep white matter of both cerebral hemispheres, relatively sparing the temporal and occipital lobes (Figure 1).

**Figure 2:**
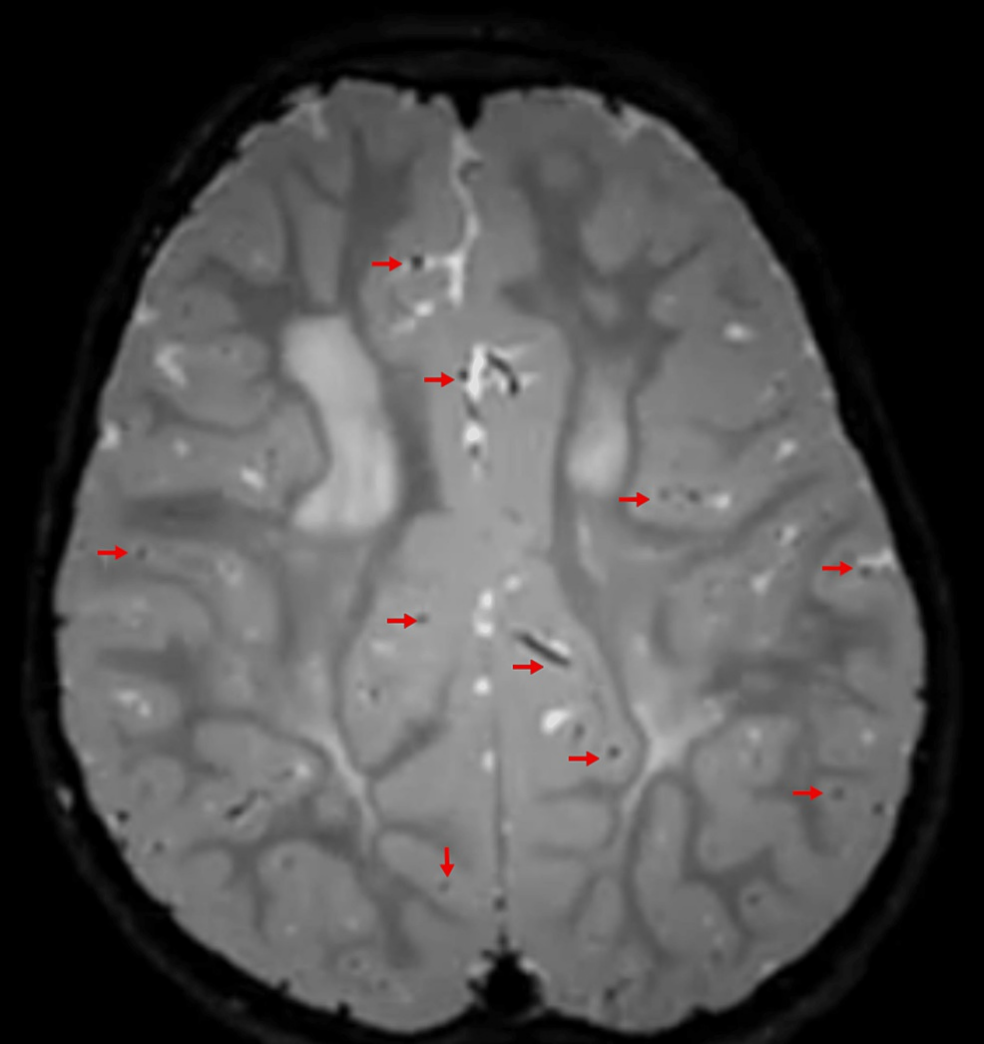
Cystic degeneration in the bi-parietal white matter (arrow heads).

Cystic degeneration was seen in the bi-parietal white matter. Relative sparing of subcortical white matter was seen. There was no post-contrast enhancement (Figure 2).

**Figure 3:**
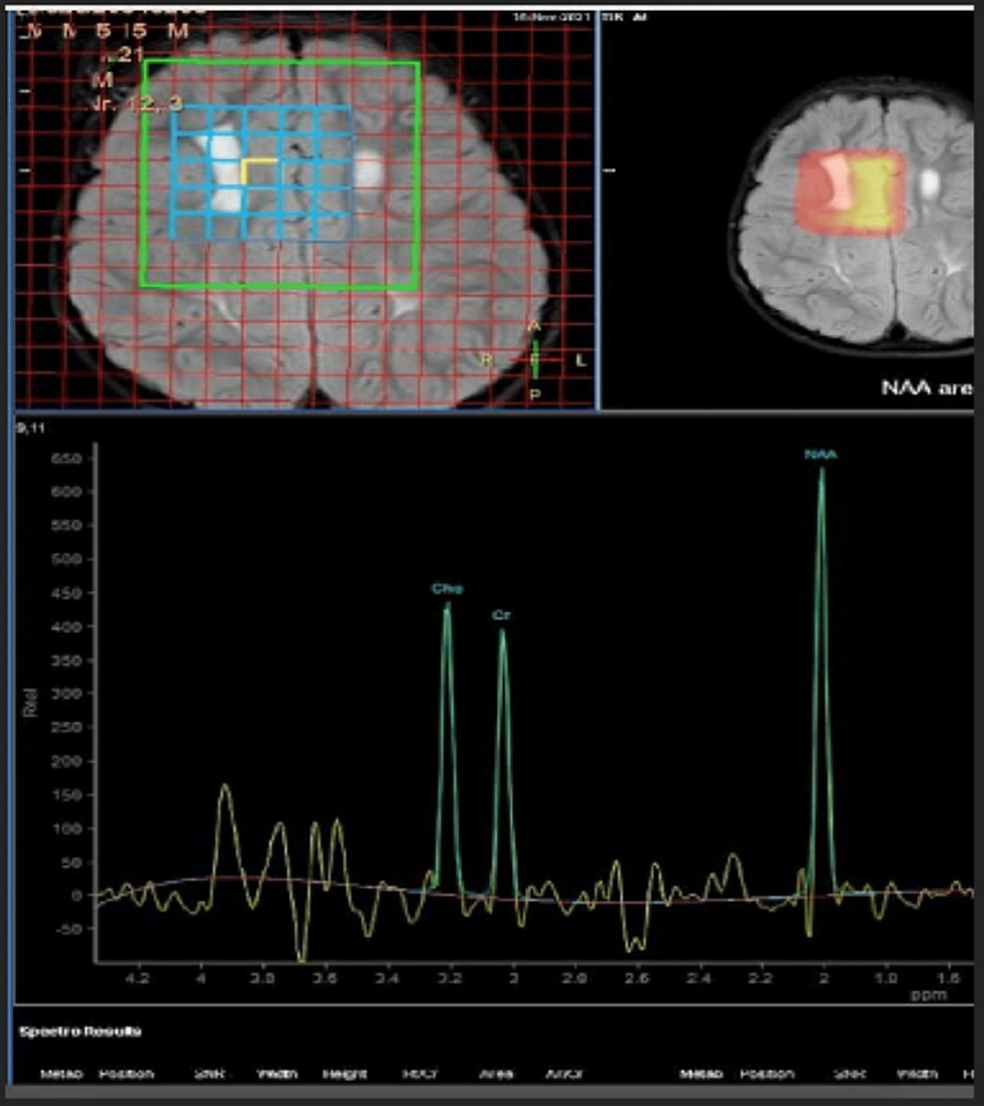
MRI spectroscopy showing signal abnormalities with lipid-lactate peak spectroscopy.

MRI spectroscopy shows elevated lipid-lactate peaks within these areas of signal change. Mild thinning of the posterior body/isthmus of the corpus callosum is noted (Figure 3).

**Figure 4:**
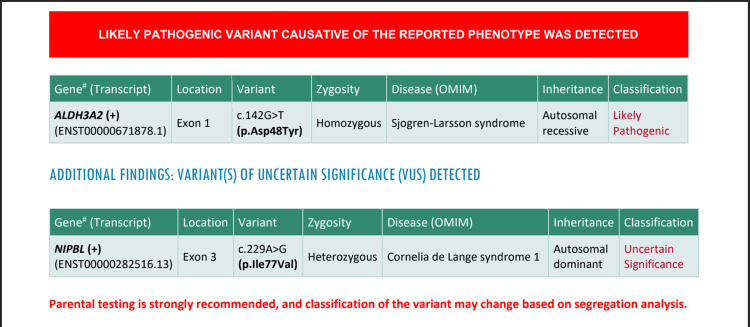
Whole exome sequencing of the patient.

All these clinical and radiological findings were diagnostic of SLS. His diagnosis was further confirmed by genetic testing, called whole exome sequencing (WES). It revealed a defect in ALDH3A2 (+) at exon 1. Findings of the test results are shown in Figure 4.

It is important to note that SLS is a chronic, progressive condition with no known cure. The treatment goal is to alleviate symptoms and improve the patient's quality of life. Our patient's treatment plan included a combination of approaches, including the use of anti-inflammatory medications such as nonsteroidal anti-inflammatory drugs (NSAIDs) and corticosteroids to reduce inflammation and pain and alleviate symptoms. For ichthyosis and pruritis, topical moisturizing creams containing urea were used, and zileuton 200 mg three times a day was given for pruritis. In addition, lifestyle changes were recommended, including drinking plenty of water to stay hydrated, using artificial tears to moisten the eyes, and avoiding dry environments and irritants that can worsen symptoms. Various forms of physical therapy to help the patient cope with the condition and improve joint mobility and flexibility, along with occupational therapy to help the patient learn to adapt to his symptoms and carry out daily activities, are being done. Further, during treatment, it was reported that the child had a low level of vitamin D, which was deduced to cause low levels of calcium absorption in the child, which in turn would have been causing weakness in muscles and nerves. At present, he walks in a wobbly manner and has been diagnosed with gait imbalance for ambulation. He has difficulty trying to stand up from a sitting position. Henceforth, he is undergoing gait and balance training and has been showing improvement. The child can run on his toes, and when not in ankle-foot orthosis (AFO), he shows a toe-walking tendency. However, he can walk on both feet slowly if coaxed to do so. He can now identify all English alphabets and numbers and can also write alphabets and numbers, but he shows signs of strain. The child is not able to concentrate on study matters; however, if concepts are communicated in short intervals and during play, the same is learned. The child can identify various colors and can correlate them with various objects. He is also able to learn new words and includes them in casual conversations with family members, though his speech is slow. He makes direct eye contact while interacting and shows enthusiasm to play with slides and other toys in the Children's Park. In his follow-up visits, the patient is also receiving counseling and psychological support to help him cope with the emotional and social impact of the condition.

## Discussion

The gene ALDH3A2 is located on chromosome 17p11.2 in the endoplasmic reticulum, which induces microsomal FALDH [6]. FALDH is responsible for fatty aldehyde and alcohol metabolism, which is necessary for cellular differentiation and function [7]. Catabolism of lipids such as fatty alcohols produces medium and long-chain aliphatic aldehydes with more than eight to 24 carbon molecules [7]. FALDH is the enzyme responsible for catalyzing the NAD+-dependent oxidation of these substrates into much smaller critical fatty acids. FALDH, along with NAD oxidoreductase, creates the fatty alcohol NAD oxidoreductase complex, which oxidizes fatty alcohols [7]. The underlying etiology of SLS is a series of mutations in the ALDH3A2 gene, resulting in the deficiency of FALDH/FAO [1]. More than 70 mutations, including amino acid substitutions, deletions, and insertions, occur in this gene and are responsible for developing SLS [5]. The deficiency of this enzyme allows the accumulation of fatty aldehydes and alcohols in cells and leads to detrimental consequences in keratinocytes and neuronal tissue [7]. SLS is inherited in an autosomal recessive pattern with 100% disease penetrance [8]. This infers that the individual affected inherits the two defective genes from both parents and experiences symptoms of the disease [9]. SLS is commonly seen in consanguineously derived offspring where both parents are from the same pedigree and are carriers of the defective gene but do not express the phenotype of the disease themselves [5]. The characteristic triad of SLS includes congenital ichthyosis, spastic diplegia or quadriplegia, and intellectual disability [1]. Ichthyosis in SLS is caused by fatty alcohol and aldehyde accumulation in the lamellar bodies of the stratum granulosum and impaired precursor transport to the stratum corneum, which interferes with skin barrier cell creation [7]. Some abnormalities found in the lamellar bodies include empty vesicles or absent vesicle membranes and fatty material accumulation in the membrane, which alters its normally stacked appearance [10]. This precipitates a hyperproliferative epidermal state with excess water loss and leads to an inability to maintain water balance, resulting in dry, itchy, scaly skin that does not shed easily [10,11]. Increased fatty alcohol and its metabolites in cells are toxic and induce cell apoptosis [3]. Some reports that focused on the neuropathological effects of SLS found that the leading causes of spastic diplegia or quadriplegia and intellectual disability were neuronal degeneration and white matter dysmyelination of the cerebral cortex and spinal tracts, specifically corticospinal and vestibulospinal [3]. Dysmyelination is defective myelinogenesis and is characterized by abnormal, arrested, or delayed myelin production, which precipitates delayed signal transmission and atrophy of the neurons in the cortex, cerebellum, and basal ganglia [12]. This presents as spastic diplegia mainly affecting the lower limbs and is characterized by gross motor developmental delays such as delay in sitting independently, crawling, or ambulation [4]. These features are accompanied by brisk deep tendon reflexes, ankle clonus, and hypertonia due to the associated spasticity [4]. Due to extensive involvement of the lower extremity causing gait imbalance, affected persons are either wheelchair-bound or are assisted with walkers or braces [13]. Intellectual deficits can range from mild to severe and progress as the child ages until puberty, with the rare possibility of cognitive deterioration occurring after the fourth decade of life [3]. Around the age of five, cognitive deficits become apparent with the exhibition of limited social interaction, delayed speech or mild aphasia, and learning difficulties. Some reports suggest that the degree of cognitive impairment directly correlates with the degree of spasticity [13].

SLS is diagnosed clinically in addition to confirmatory imaging with CT and MRI of the brain. MRI of the brain with T2 and fluid-attenuated inversion recovery (FLAIR) images shows leukoencephalopathy with zones of increased signaling abnormalities within the periventricular white matter, corona radiata, parietal lobes, and corpus callosum. T1 images show hypointense regions of delayed signal intensity affecting both cerebral hemispheres [5]. The subcortical white matter and temporal and occipital lobes are relatively spared with preferential involvement of the frontal or parietooccipital zones, which manifested as cystic degeneration of white matter in our case [3]. Another test that can be utilized with a pathognomonic finding is MRI spectroscopy. An abnormal lipid peak resonance of 1.3 ppm within the areas of T2 cerebral white matter changes is specific to SLS. It indicates that there is an accumulation of fatty alcohols and aldehydes in these areas [14]. SLS is treated symptomatically with emollients and keratolytic for ichthyosis and speech and physical therapy for developmental and motor delays [15]. Implementing a low-fat diet with a supplemental intake of medium-chain fatty acids has allowed symptomatic improvement in persons with SLS [3]. It has been reported in clinical studies that the drug Zileuton has successfully reduced the intensity of pruritis in those affected. Leukotriene B4 (LTB4) is a proinflammatory agent that is catabolized by a FALDH-dependent pathway. It is suspected to be an alternative underlying cause of pruritis in SLS [16]. Zileuton is a leukotriene synthesis inhibitor that decreases the concentration of LTB4 and subsequently improves the symptoms of pruritis in persons affected [16]. In our case, the patient was the product of a consanguineous relationship, increasing the probability of inheriting this disorder. His symptoms consisted of congenital ichthyosis, developmental delay, and spastic limb diplegia with involvement of the lower extremity within the first decade of life. The pathognomic finding of elevated lipid peak resonance was discovered on MRI spectroscopy, confirming his diagnosis. The patient has been undergoing symptomatic treatment with moderate improvement in his symptoms. SLS should be considered in persons with this characteristic triad of ichthyosis, spastic limb diplegia, and developmental delay. A high degree of suspicion is required by physicians when these symptoms are present, so diagnostic tests with brain MRI and MRI spectroscopy and early intervention with dietary changes, medications, and speech and physical therapy can be implemented to prevent the progression of this disorder and increase the quality of life in adolescents.

## Conclusions

SLS is a rare genetic disorder that arises in adolescents due to the autosomal recessive deficiency of FALDH. Consanguineous relationships remain the number one risk factor for developing this disease, and patients clinically exhibit symptoms within the first decade of life. The underlying cellular toxicity and apoptosis lead to the inability to maintain the skin epidermal water balance and dysmyelination of the cerebral cortex and spinal tracts resulting in the triad of congenital ichthyosis, spastic diplegia, and intellectual disability. The presence of clinical features and imaging with gold-standard MRI spectroscopy allows physicians to establish this diagnosis. This condition necessitates a high index of clinical suspicion when presented with this triad of symptoms in adolescents. Early intervention with lifestyle changes and symptomatic management is required to decrease the severity of this disorder and allow patients to maintain an optimal quality of life.
